# A Semantic Segmentation Method Based on AS-Unet++ for Power Remote Sensing of Images

**DOI:** 10.3390/s24010269

**Published:** 2024-01-02

**Authors:** Guojun Nan, Haorui Li, Haibo Du, Zhuo Liu, Min Wang, Shuiqing Xu

**Affiliations:** School of Electrical Engineering and Automation, Hefei University of Technology, Hefei 230009, China; zzbngj@hfut.edu.cn (G.N.); haorui.li@mail.hfut.edu.cn (H.L.); zhuo.liu@mail.hfut.edu.cn (Z.L.); 2020170397@mail.hfut.edu.cn (M.W.); xsqanhui91@gamil.com (S.X.)

**Keywords:** semantic segmentation, Unet, atrous spatial pyramid pooling, squeeze-and-excitation module

## Abstract

In order to achieve the automatic planning of power transmission lines, a key step is to precisely recognize the feature information of remote sensing images. Considering that the feature information has different depths and the feature distribution is not uniform, a semantic segmentation method based on a new AS-Unet++ is proposed in this paper. First, the atrous spatial pyramid pooling (ASPP) and the squeeze-and-excitation (SE) module are added to traditional Unet, such that the sensing field can be expanded and the important features can be enhanced, which is called AS-Unet. Second, an AS-Unet++ structure is built by using different layers of AS-Unet, such that the feature extraction parts of each layer of AS-Unet are stacked together. Compared with Unet, the proposed AS-Unet++ automatically learns features at different depths and determines a depth with optimal performance. Once the optimal number of network layers is determined, the excess layers can be pruned, which will greatly reduce the number of trained parameters. The experimental results show that the overall recognition accuracy of AS-Unet++ is significantly improved compared to Unet.

## 1. Introduction

Recently, how to automatically plan transmission lines for power has attracted many researchers. Based on geographic information, such as houses, roads, forests, rivers, etc., selection algorithms can plan a reasonable transmission line. Hence, accurate geographic information is essential for the automatic construction of transmission lines [1,2]. With the progress of satellite technology, the image resolution acquired by remote sensing satellites is constantly improving. The different types of feature information in remote sensing images can satisfy the requirements for the planning of transmission lines [3]. In the literature, the methods for feature recognition from remote sensing images can be divided into two kinds. One is based on the classical image threshold segmentation technology and the manual map marking. The other method is called image semantic segmentation, which is based on deep learning.

The traditional image segmentation method is based on the color, shape, texture, and other features of the image, which leads to the image content being divided into different regions according to the edge features [4,5,6]. The classification results can be optimized to some extent by extracting geometric information from the image and combining it into class-by-class pixels [7]. However, the traditional image segmentation method can only split the target and background of the image [8], which ignores other feature information from remote sensing images [9].

With the development of computer technology, using deep learning for image recognition and semantic segmentation has become a research hotspot. Deep learning technologies continue to advance, and existing methods are constantly optimized and improved, gradually improving performance and robustness. Pedestrian recognition in videos is interfered with by many factors, such as environmental changes, occlusions, and so on. The adaptive interference elimination framework has been adopted to model the motion path of each pedestrian in recent work, which can effectively solve these interference problems [10]. Capturing detailed and informative features from the input images, utilizing pixel-level supervision to learn discriminative feature representations for forest smoke recognition [11], introducing label relevance and multi-directional interactions to improve the recognition accuracy, and using enhanced deformable convolution to extract more accurate feature representations [12] has enhanced the recognition ability of forest smoke, effectively warning of a forest fire.

With the progress of satellite technology, the image resolution of remote sensing satellites is gradually increasing. In this case, with the development of deep learning and graphics processing units [13,14], the semantic segmentation of remote sensing images has become a hotspot in the study of transmission line planning [15]. Semantic segmentation based on full convolutional neural networks (FCNN) can satisfy input images with arbitrary size [16]. By modifying the FCNN, many different semantic segmentation networks have been proposed in the literature, such as SegNet [17,18], Unet [19], and Deeplab [20]. Although the segmentation accuracy of the target is improved, to some extent, the detailed feature processing is still unsatisfactory. For example, in the structure of Deeplabv3+, the down-sampling operation leads to the loss of some of the image information [21], which results in low recognition accuracy of the small targets in high-resolution semantic segmentation.

Semantic segmentation has been widely applied in power line construction. Using semantic segmentation to identify power equipment in high-voltage transmission lines and substation scenarios can realize automatic inspection of power systems. Detecting power transmission infrastructure from aerial images using deep learning [22] and identifying objects like buildings, roads, forests, and rivers in remote sensing images using neural networks [23] provides effective information for transmission line planning. However, there is a relative lack of research on using neural networks to identify and segment object elements in transmission line planning, and the neural networks used are mostly basic ones, which have significant drawbacks. Improving neural networks and accurately identifying objects like buildings, roads, forests, and rivers in transmission line planning is of great significance.

Unet is an improved neural network based on (FCNN) [24]. Compared with FCNN, Unet has higher sensitivity to image details, higher processing accuracy, and the ability to realize spatial consistency by considering pixel-to-pixel relationships. However, in the structure of Unet, due to the down-sampling methods of using convolution with step size and pooling operations, there is a loss of targeted and detailed spatial information. Hence, it is not ideal for the extraction of targets with a small pixel share in remote sensing images. In addition, the Unet structure is a neural network with a fixed number of layers, which can only extract features with a fixed depth [25]. That is to say that it is difficult to extract features with different depths and shades of information in remote sensing images.

To conquer the down-sampling problem of Unet, in this paper, we firstly add the ASPP (atrous spatial pyramid pooling) and the squeeze-and-excitation (SE) module [26] to Unet such that the sensing field can be expanded and important features can be enhanced. This improved Unet is called AS-Unet in this paper. Secondly, note that the information features contained in remote sensing images are of different depths and the number of layers of the network have different performances for the features of different depths. Meanwhile, the selection of the number of layers will have a great impact on the performance of image segmentation. To solve the problem, this paper combines AS-Unet with a different number of layers in accordance with the network structure of Unet++, which is called AS-Unet++. The advantage of an improved network is that it can automatically determine an optimal performance depth for different depth features. Once the optimal number of network layers is fixed, the redundant layers can be pruned, which greatly reduces the number of trained parameters. Finally, by comparing and analyzing the experimental data, the trained AS-Unet++ shows better recognition accuracy in the semantic segmentation of remote sensing images compared to Unet. It will provide reliable remote sensing image data for the automatic planning of transmission lines.

## 2. AS-Unet++ for Remote Sensing of Images

In this section, we introduce the main construction process of the proposed AS-Unet++.

In practice, there are two main factors that affect image segmentation accuracy. One is that the feature information from remote sensing images has different depths. The other one is that the feature distribution is not uniform, and the proportion of different factors is not the same. To this end, our proposed AS-Unet++ is built with the following steps.

**Step 1: design of AS-Unet.** Add the ASPP and SE modules to Unet such that the sensing field can be expanded and important features can be enhanced.

**Figure 1:**
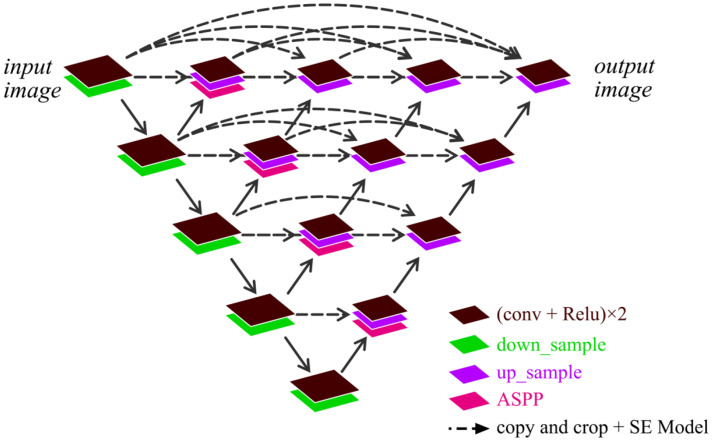
AS-Unet++ structure.

**Step 2: construction of AS-Unet++.** Since the choice of the number of layers has a great impact on the performance of image segmentation, the Unet++ is a network structure that combines and connects 2 to 5 layers of Unet; compared to Unet, Unet++ allows the network to automatically learn features of different depths and determine a depth with optimal performance. Based on the same idea as the structure of Unet++, two to five layers of AS-Unet are combined such that each AS-Unet shares a common left feature extraction part. Each layer of AS-Unet is a Unet that incorporates the ASPP with the SE Model, which becomes the AS-Unet++ studied in this paper. It is shown in Figure 1.

Next, we will give a detailed introduction to this new network structure.

### 2.1. Unet

**Figure 2:**
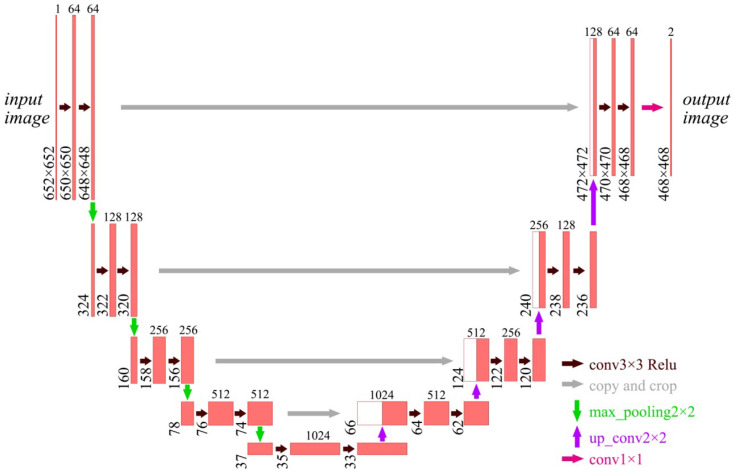
Unet structure.

Unet is an improved network of FCNN [27], and the structure is a symmetric U-shaped structure. It mainly consists of two parts: feature extraction and feature enhancement, which is shown in Figure 2. The feature extraction part is on the left side and the feature strengthening part is on the right side. It has five layers.

The number above each layer in Figure 2 is the number of feature layers contained in that layer, and the number to the left of each layer is the size of the layer.

(1) **The part of feature extraction**

Different layers are connected to each other with a 2 × 2 max-pooling layer, which is labeled by the green arrow in Figure 2. The size of the max-pooled image is halved every time it passes through the max-pool. Since the padding is not set, some feature information is lost if the size is odd. Therefore, it is required to carefully set the size of the input image and keep the image length and width as an even number of pixel points. With the increasing number of channels in the convolutional layer, the number of feature channels in the image will increase.

(2) **The feature enhancement**

The layers are connected by a 2 × 2 inverse max-pooling up-sampling layer, which is labeled by the purple arrow in Figure 2. The size of the image will be doubled after each up-sampling. Each layer of the feature enhancement network will fuse the features from the left feature extraction part, which is shown by the gray arrow. However, the image on the left side is larger than that on the right side, so some shearing is needed before feature fusion.

Each layer between the two parts will conduct a 3 × 3 convolution operation, which is labeled by the brown arrow in Figure 2. Then, it is followed by a Relu activation function layer. The operation mode of the convolution operation is a valid mode where the stride is 1 and the convolution kernel is 3 × 3. Since the padding is also not set, the image size will be reduced by two after each convolution operation. The last layer of the output is classified by a 1 × 1 convolution layer, which is shown by the magenta arrow in Figure 2.

In the original structure of Unet, the down-sampling methods, such as convolution and pooling with step size, will lead to the loss of target spatial information and detailed features. Hence, the extraction of targets with small pixel proportions in remote sensing of images is not ideal. The Unet structure is a neural network with fixed layers (usually five layers) and can only extract features of a fixed depth. Therefore, it is difficult to adapt to the information features of different depths in remote sensing images.

### 2.2. AS-Unet

#### 2.2.1. AS-Unet Structure

**Figure 3:**
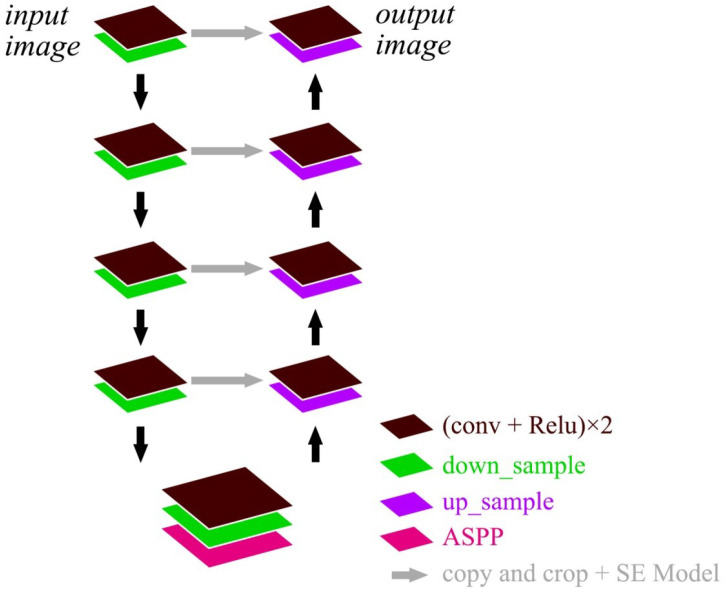
AS-Unet structure.

Remote sensing images contain complex feature information, the feature distribution is not uniform, and the proportion of different factors is not the same. In order to solve the problems in the original Unet structure that down-sampling methods create, such as convolution and pooling with step size, which lead to the loss of targeted spatial information and detailed information, the ASPP and SE models are added to Unet. This is called AS-Unet, which expands the sensing field such that the loss of information can be reduced and the important features can be enhanced. The structure of AS-Unet is shown in Figure 3.

The SE model is added to the feature fusion part of each layer, which can automatically evaluate the importance of each feature channel. Different weight coefficients can be added to each channel such that important features are strengthened and unimportant features are suppressed. After the convolution and activation function in the last layer of the network, adding an ASPP module can expand the sensory field such that information loss can be minimized and the network’s ability to capture multi-scale information is improved.

#### 2.2.2. Atrous Spatial Pyramid Pooling (ASPP)

**Figure 4:**
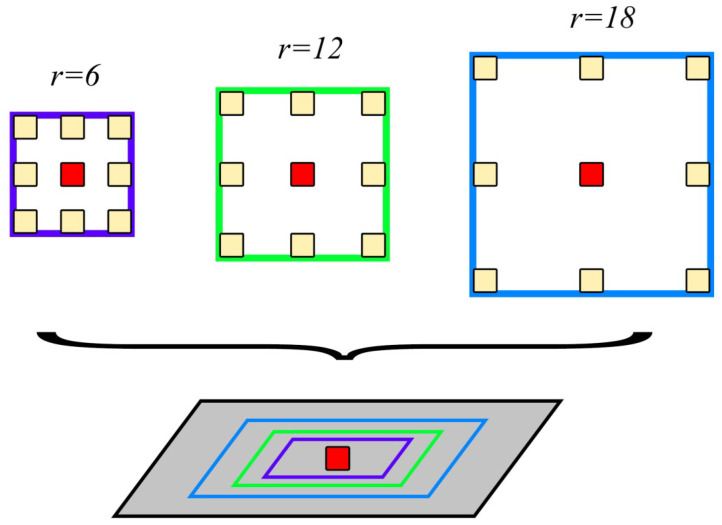
ASPP structure.

ASPP uses parallel null convolutional layers with multiple different sampling rates, where the features extracted for each sampling rate are further processed in separate branches and fused to generate the final result. This expands the sensor field while ensuring that the resolution is not degraded by the Unet down-sampling operation. Meanwhile, it enhances the ability to capture multi-scale contextual information. Figure 4 illustrates the specific structure of ASPP. The *r* = 6, 12, and 18 in Figure 4 represent convolution kernels with null rates of 6, 12, and 18, respectively.

ASPP constructs convolutional kernels with different receptive fields by different atrous rates and obtains the multi-scale contextual information through parallel structures [28]. The information of different scales is integrated by the concat method [29]. The structure can be given as:

(1) $$O\left[j\right]=\sum_{f}I\left[j+r•n\right]f\left[n\right],$$

where O[j] is the output of the convolution operation performed on the pixel with index *j*, *I* is the input feature mapping, *r* is the atrous rate of the convolution kernel, *f* is the convolution kernel with weights, and *n* is the convolution kernel position index.

The ability to change the receptive field (RF) size by varying the value of the cavitation rate *r* is calculated as follows:

(2) $$\mathrm{RF}=\left(2^{r+1}-1\right)\times \left(2^{r+1}-1\right)$$

where *r* is usually chosen to be 6, 12, or 18. Too large of a value will lead to too sparse sampling of the input signal, which results in no correlation between the remote information.

#### 2.2.3. Squeeze-and-Excitation (SE)

The feature distribution of remote sensing images is not uniform. If the house elements are divided, the training effect of this part of the training set will be poor due to the small proportion of house elements in some training sets.

**Figure 5:**
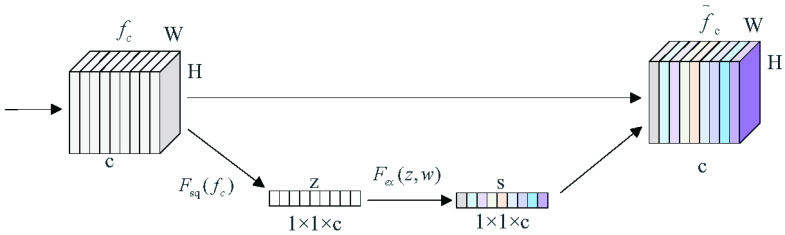
SE structure.

The squeeze and excitation can utilize the relationship between different channel feature mappings such that the specific semantic features can be strengthened [30]. The SE module can assign different weights to each channel, which means that channels containing important information features are strengthened and the channels with non-important information features are weakened [31] to optimize the training effect. The SE structure is shown in Figure 5.

The SE module contains two main operations, squeeze and excitation, which are $F_{s}q$ and $F_{e}x$ in Figure 5. $f_{c}$ is the feature map with feature channel *c*, H is the height of the feature map, and W is the width of the feature map. *z* is the feature vector of 1 × 1 × *c*, *s* is the weight, and ${\tilde{f}}_{c}$ is the weighted feature channel. The compression operation is represented by the following equation:

(3) $$z=F_{sq}\left(f_{c}\right)=\frac{1}{h•w}\sum_{i=1}^{h}\sum_{j=1}^{w}f_{c}\left(i,j\right),$$

where $f_{c}$ is the feature map with feature channel *c*, *h* is the feature map height, and *w* is the feature map width.

The squeeze operation can be performed by global average pooling based on the width and height of the feature maps, and the scalar that represents the global receptive field [32]. The feature maps with dimensions w×h×c, which contain the global information, are compressed into 1×1×c feature vectors *z*, such that the generated channel statistic *z* contains contextual information. It can alleviate the channel dependency problem.

The excitation operation is accomplished by fitting the nonlinear relationship between the channels through two fully connected layers and an activation function. To reduce the computational effort, the first fully connected layer compresses the *c* channels and then a Relu function is used as the activation function. The second fully connected layer restores the number of channels to *c*, and the weights *s* are obtained by activating the Sigmoid activation function. *s* is calculated as follows:

(4) $$s=F_{ex}\left(z,\omega \right)=\sigma \left(h\left(z,\omega_{1}\right)\right)=\sigma \left(\omega_{2}\delta \left(z•\omega_{1}\right)\right),$$

where ω represents the parameters of the fully connected layer, δ represents the Relu activation function, and σ represents the sigmoid activation function.

The original channels are weighted by using the obtained weights, *s*. Valid feature channels have larger weights and invalid or unimportant feature channels have smaller weights.

### 2.3. Unet++

#### 2.3.1. Unet with Different Depths for Each Layer in Unet++

The feature information contained in remote sensing images is rich and the distributed categories are not uniform, which means that the information is characterized by different depths. Unet models of different depths will have different performances.

**Figure 6:**
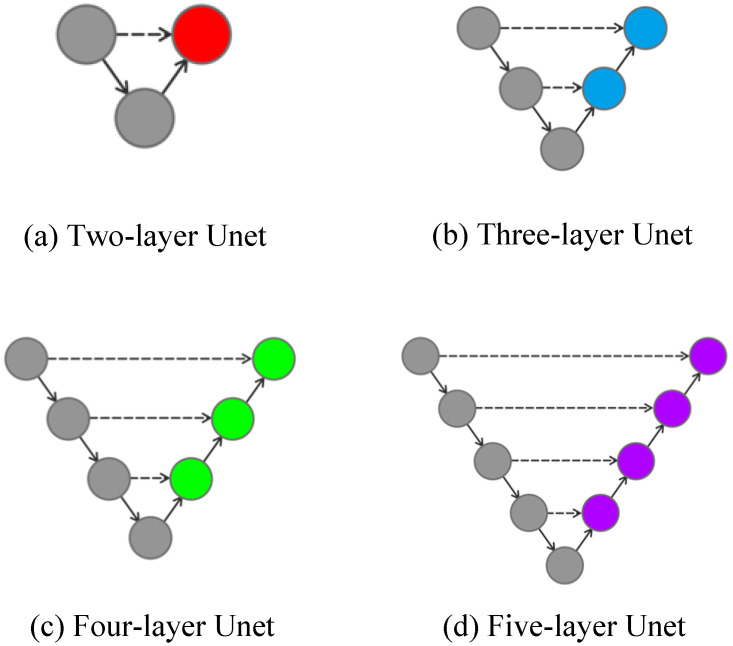
Unet with different depths.

Unet++ is a network structure connected by a combination of different layers of Unet, and different layers of Unet can realize the extraction of different depth features. Figure 6 shows the Unet structure with different depths.

**Figure 7:**
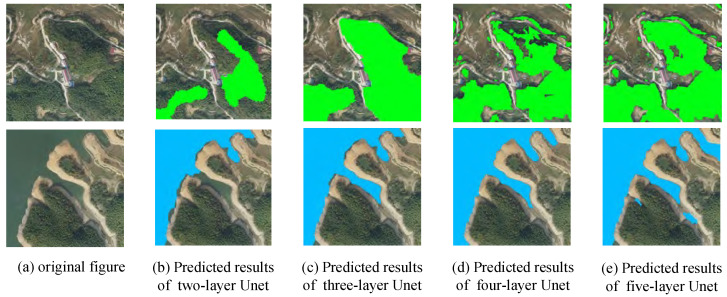
Predicted results of Unet with different depths.

Forest element segmentation and lake element segmentation are performed on the two remotely sensed images using different depths of Unet. The segmentation results are shown in Figure 7.

**Figure 8:**
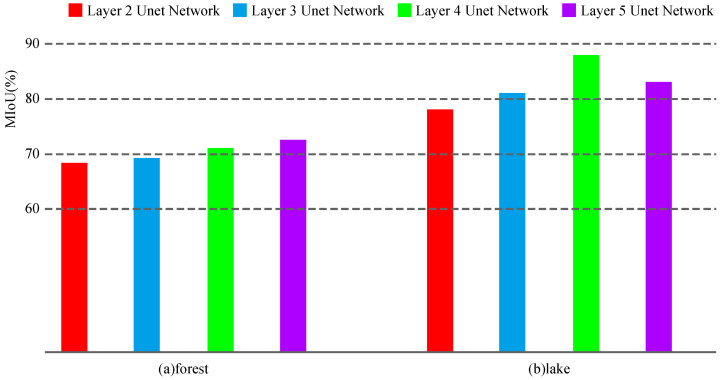
Predicted MIoU for Unet of different depths.

The MIoU obtained from the results of Figure 7 is shown in Figure 8.

As can be seen from Figure 8, the segmentation performance for forests can be improved by adding the number of network layers. However, for lakes, the Unet with four layers performs better than the Unet with five layers.

In the recognition of forest elements, there is a serious leakage of recognition in the shallow Unet, and there is also misrecognition of non-forest elements in the Unet of layers 2 and 3. As the number of network layers increases, the phenomenon of missed recognition becomes less and less. The 5-layer Unet basically alleviates the phenomenon of missed recognition and misrecognition. In the recognition of lake elements, the Unet of layers 2 and 3 has the phenomenon of missed recognition. The Unet of layer 4 can recognize the lake elements completely and accurately. However, the Unet of layer 5 has the phenomenon of misrecognition of non-lake elements similar to the contours of a lake with an increased number of layers. From the above analysis, it can be seen that remote sensing images contain complex and rich information; the feature depth of the information is not the same. The number of layers of the network will also have different performances for features with different depths. An increase in the number of layers of the network may not necessarily represent an improvement in the recognition performance.

#### 2.3.2. Unet++ Structure

**Figure 9:**
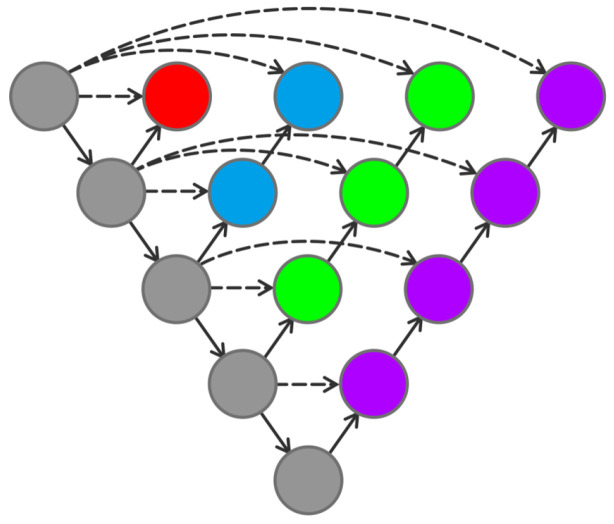
Combination of Unet with different depths.

The choice of the number of Unet layers has an important impact on the performance of image segmentation. By combining the different layers of Unet, as shown in Figure 9, different levels of features can be captured.

**Figure 10:**
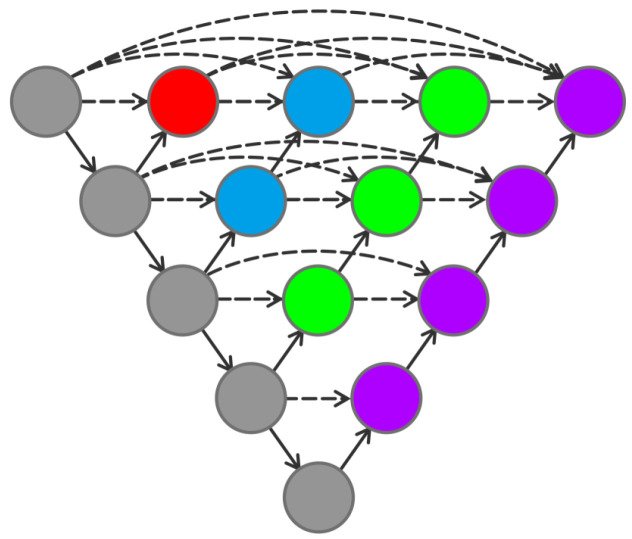
Unet++ structure.

However, because of the lack of connections in the middle region of the network structure, the gradient cannot pass through this region. It implies that the backpropagation breaks down here and the network cannot be trained. By adding connections to the middle units, the problem can be solved. Connecting all the middle units is the structure of Unet++, which is shown in Figure 10.

From Figure 10, Unet++ is a network structure that combines 2 to 5 layers of Unet together. Compared to Unet, Unet++ allows the network to automatically learn features at different depths and determine a depth with optimal performance. Once the optimal number of network layers is determined, the excess layers can be pruned, which greatly reduces the number of trained parameters.

In addition, it can be seen from Figure 10 that the Unet feature extraction parts of each layer are superimposed together. That is to say that the Unet ++ allows different levels of Unet to share a left feature extraction unit, which reduces the training amount of multiple Unets.

### 2.4. AS-Unet++

AS-Unet++ is based on the Unet++ structure, which combines an AS-Unet of 2 to 5 layers. Each AS-Unet shares a common left feature extraction part. Each layer of AS-Unet is a Unet that joins ASPP with SE Model; its structure is shown in Figure 1.

Compared to Unet, due to the use of ASPP and SE models, AS-Unet++ enhances the ability of neural networks to extract important feature information and capture multi-scale contextual information. AS-Unet++ allows the network to automatically learn features at different depths and determine an optimal performance depth. Once the optimal number of network layers is fixed, the excess layers can be pruned to reduce the number of parameters to be trained. When the AS-Unet feature extraction portions of each layer are stacked together, the AS-Unet of different layers share a common left-side feature extraction unit, which reduces the amount of training for multiple AS-Unets.

## 3. Experiments

### 3.1. Data

The image data used in this paper are taken from high-resolution remote sensing images of Fuyang City, Anhui Province, China. The image size is cut to 4000 × 4000 with 160 images in total, and vector semantic segmentation labels are made using QGIS software (Version 3.10, Gary Sherman, Cedar Rapids, USA). Then, the remote sensing image is cropped into 300 × 300 deep learning samples by the sliding window cropping method. The categories of feature information in the remotely sensed images are houses, roads, forests, and lakes.

All the data are divided into two sets, training set and validation set, and the ratio of training data to validation data is 4:1. The training set images are fed into the network for training, and then the validation set is fed into the trained network for prediction to evaluate the results and performance of the training.

**Figure 11:**
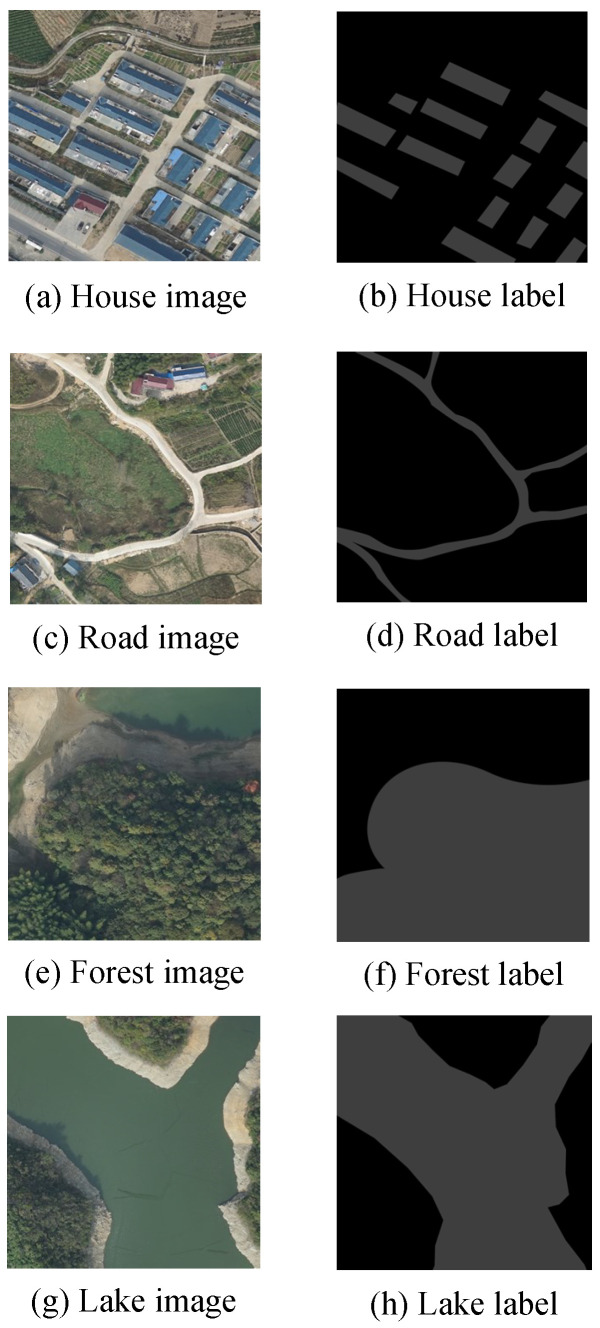
Remote sensing images and labels.

The labels of the images are created using QGIS, and different shades of gray are used to refer to different things in the image. Pixels of the same type of things are labeled with a fixed gray value, and the background is solid black. Figure 11 shows the images of houses, roads, forests, and lakes along with the labels.

By randomly flipping the training data horizontally, vertically, diagonally, and with appropriate linear stretching, the generalization ability of the model can be enhanced, and the number of data sets can be expanded. In addition, blurring the image and adding noise can prevent the model from learning unnecessary noise and inhibit overfitting. Among them, 15% of the images were randomly rotated by 90°, 5% were flipped horizontally, 5% were flipped vertically, and 10% had blur and noise added to them. After cropping and data enhancement, a total of 12116 remote sensing images with 300×300 resolution were obtained as training data. To simulate the variable domain, the validation dataset is linearly stretched by 0.8%, 1%, 1.5%, and 2% at random.

### 3.2. Environment and Parameter Configuration

The environment configuration for AS-Unet++ is shown in Table 1.

The training parameters used in the experiment are shown in Table 2.

### 3.3. Evaluation Indicator

The evaluation indicators are Precision, Recall, IoU, and MIoU. The evaluation indicators are calculated on the basis of a confusion matrix, as shown in Table 3.

Precision represents the proportion of correctly predicted pixels in a certain category, its calculation formula is as follows:

(5) $$\;\mathrm{Precision}\;=\frac{TP}{TP+FP}$$

Recall represents the proportion of the total number of pixels in a certain category that have been correctly recognized by the network, the calculation formula of which is as follows:

(6) $$\;\mathrm{Recall}\;=\frac{TP}{TP+FN}$$

IoU is the ratio of the intersection and union between the predicted result and the true value of each class. MIoU can be obtained by adding the intersection ratio of each class to the average value. The larger the MIoU, the closer the predicted result is to the true value [33]. The IoU and MIoU are calculated as follows:

(7) $$\begin{array}{c} \;IoU=\frac{TP}{TP+FP+FN},\\ MIoU=\frac{\sum IoU}{n}, \end{array}$$

where *n* is the number of categories for image segmentation.

### 3.4. Experimental Results

In order to better evaluate the performance of the network semantic segmentation method based on AS-Unet++ proposed in this paper, we conducted three sets of experiments. The first experiment was an ablation experiment, where AS-Unet++, Unet++, A-Unet++ with only ASPP added, and S-Unet++ with only the SE model added are compared with each other in the test set to verify the effectiveness of the two modules, the ASPP and SE models. The second experiment is to compare AS-Unet++ with Unet and AS-Unet model in the training set and test set, which can visualize the performance optimization of the network. The third experiment compared AS-Unet++ with other network models in the training and test sets, including CE Loss and regular data enhancement, to evaluate the method of this paper through further comparative experiments.

(1) **The ablation experiment**

AS-Unet++ and Unet++ were compared with A-Unet++ with only ASPP added and S-Unet++ with only the SE model added to validate the effectiveness of the two modules of the ASPP and SE model.

**Figure 12:**
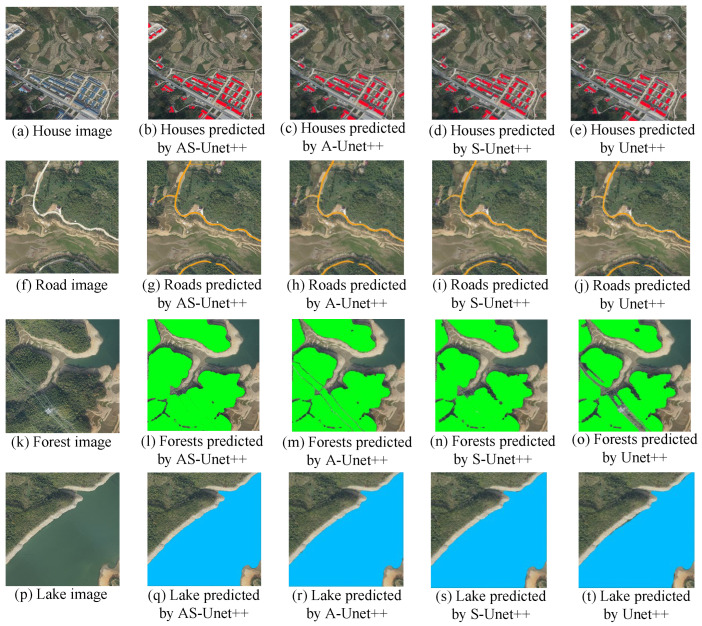
Predicted segmentation images for the ablation experiment.

A comparison of the predicted segmentation maps for houses, roads, forests, and lakes realized by various networks is shown in Figure 12.

In the recognition of house elements, Unet++ has the phenomenon of missed recognition of some houses due to the difference of light and color, and the edge segmentation effect of houses is not good in recognition. In A-Unet++ with only the addition of ASPP, although the edge segmentation effect of houses has been improved, the phenomenon of missed recognition has not been improved. In S-Unet++ with only the SE model, although the phenomenon of missed recognition has been improved, the edge segmentation effect of houses has not been improved. The AS-Unet++ network with the addition of both modules improved in both missed recognition and the edge segmentation effect. Compared with A-Unet++ with the addition of a single module, it was not improved in the recognition of houses. In S-Unet++ with only the SE model, although the omission recognition phenomenon was improved, the edge segmentation effect of houses was not improved.

AS-Unet++ with both modules improved in both omission recognition and edge segmentation effects, and the improvement was more obvious compared with A-Unet++ and S-Unet++ with a single module. In remote sensing images, the house element accounts for a relatively small proportion, and the lack of SE model has poor performance in capturing semantic features of the house, which leads to the phenomenon of missed recognition. While the lack of ASPP leads to the loss of target spatial information and detailed information caused by downsampling methods, such as convolution and pooling with step size in the original structure of the Unet, it does not have much effect on the house information, such as illumination and color differences, but causes the edge feature information to be lost. Although it does not have much effect on the information of light and color differences in the house, it will cause the loss of edge feature information, resulting in an unsatisfactory edge segmentation effect.

In the recognition of road elements, Unet++ has the phenomenon of missing recognition of some roads due to small widths, and the edge segmentation effect is also poor. A-Unet++ improves the edge segmentation effect of roads, but the phenomenon of missing recognition remains unimproved. S-Unet++ improves the phenomenon of missing recognition, but the edge segmentation effect has not been improved. AS-Unet++ improves the phenomenon of missing recognition in both aspects. Similar to the house element, the road element occupies a relatively small proportion in remote sensing images, and the lack of SE model results in poor capture of road information, which leads to the phenomenon of missed recognition. The lack of ASPP results in the loss of edge feature information, which leads to unsatisfactory edge segmentation effects.

In the recognition of forest elements, Unet++ recognizes the surrounding forest pixels poorly due to the interference of the wire pixels in the lower right corner. S-Unet++ completely recognizes the wire pixels as forest pixels compared with Unet++, with no significant improvement in the recognition performance. A-Unet++ can split the wire pixels and the forest pixels better than Unet++, and the recognition effect is closer to that of AS-Unet++. The lack of SE model does not affect the network’s ability to capture forest information in remote sensing images because the forest elements account for a large proportion of the remote sensing image. The interference of power lines crossing from the forest in remote sensing images become elements with a small proportion in the remote sensing image, and the lack of ASPP results in the loss of interference information, which in turn leads to poor anti-interference ability.

In the recognition of lake elements, the recognition performance of Unet++ and S-Unet++ is similar, and the recognition in the edge part is not satisfactory enough. The recognition performance of A-Unet++ and AS-Unet++ is similar, and both of them improve in the recognition of edges. Same as the forest element, the lake element occupies a relatively small proportion in the remote sensing image, and the lack of SE model does not affect the network’s ability to capture the lake information. While the lack of ASPP leads to the loss of edge feature information, which in turn leads to an unsatisfactory effect of edge segmentation.

The Precision, Recall, and IoU of various networks for house, road, forest, and lake predictions in the test sets are shown in Table 4.

The MIoU of AS-Unet++ on the test set was 90.2%. Meanwhile, Unet++ had 83.2% MIoU for the test set, A-Unet++ had 86.6% MIoU for the test set, and S-Unet++ had 86.2% MIoU for the test set. Compared with Unet++, A-Unet++, and S-Unet++, the MIoU of AS-Unet++ was improved by 7.0%, 3.6%, and 4.0%, respectively.

In the identification of house elements, AS-Unet++ improved the three metrics of Precision, Recall, and IoU by 5.8%, 6.4%, and 6.3%, respectively, compared to Unet++, and the three metrics of A-Unet++ improved by 4.9%, 4.4%, and 4.5%, respectively, compared with S-Unet++, and the three metrics of S-Unet++ improved by 2.9%, 2.3%, and 2.8%, respectively.

In the identification of road elements, AS-Unet++ improved 6.4%, 7.0%, and 6.9% in the three metrics compared to Unet++; 3.3%, 2.1%, and 2.9% in the three metrics compared to A-Unet++; and 2.4%, 1.3%, and 2.0% in the three metrics compared to S-Unet++.

In the identification of forest elements, AS-Unet++ improved 9.7%, 9.3%, and 9.5% in the three metrics compared to Unet++; 5.4%, 4.6%, and 5.2% in the three metrics compared to A-Unet++; and 7.6%, 5.7%, and 6.7% in the three metrics compared to S-Unet++.

In the identification of lake elements, AS-Unet++ improved the three metrics by 5.5%, 5.4%, and 5.4%, respectively, compared to Unet++; improved the three metrics by 2.5%, 1.2%, and 1.8%, respectively, compared to A-Unet++; and improved the three metrics by 5.0%, 3.9%, and 4.3%, respectively, compared to S-Unet++.

In the recognition of house elements and road elements, S-Unet++ was higher compared to A-Unet++, which shows that the SE model improves the performance of recognition of elements with smaller pixel occupancy more significantly. In the recognition of forest elements and lake elements, A-Unet++ was higher compared to the three metrics of S-Unet++, and ASPP had better recognition performance in the recognition of elements with large pixel occupancy because of better edge segmentation and better resistance to interference with small occupancy.

(2) **Comparison of AS-Unet++, Unet, and AS-Unet**

Comparing AS-Unet++ with Unet and the AS-Unet model in the training sets and test sets allows for visualization of the performance optimization of the network.

**Figure 13:**
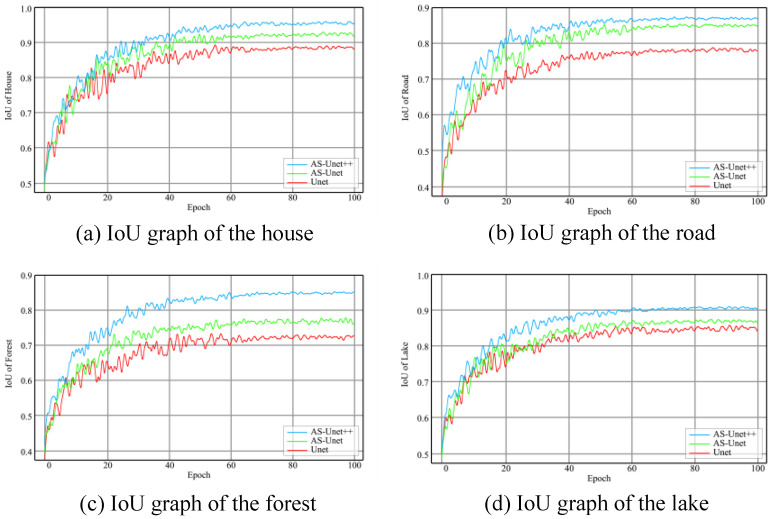
IoU graphs during training of AS-Unet++, Unet, and AS-Unet.

The graphs of MIoU in the three kinds of networks during the training of houses, roads, forests, and lakes are shown in Figure 13.

It can be seen that after training, the MIoU of the AS-Unet++ verification set reached 88.9%. However, the MIoU of Unet and AS-Unet on the verification set was 80.8% and 85.8%, respectively.

The Precision, Recall, and IoU of the verification sets of each network for road elements, forest elements, and lake elements are shown in Table 5.

It can be seen from the above data that the AS-Unet network is superior to the Unet network in all indicators, and the AS-Unet++ network, as a further optimization of the AS-Unet network, has improved in all aspects of accuracy compared with the AS-Unet network.

Compared with the AS-Unet and Unet network, the MIoU of the AS-Unet++ network increased by 3.1% and 8.1%, respectively. In Figure 13, the overall convergence speed of the three kinds of differences was small, and, only in the road elements recognition training, the AS-Unet++ network convergence speed was slightly faster than the other two networks. In addition, in the training process, AS-Unet++ compared with the other two network oscillations was smaller, especially in the roads, forests, and lakes element recognition training. In the identification of house elements, the Precision index increased by 2.7% and 6.4%, the Recall index increased by 3.2% and 7.4%, and the IoU index increased by 3.1% and 7.3%, respectively. In the recognition of road elements, the Precision index increased by 2.5% and 9.3%, Recall increased by 2.1% and 9.6%, and IoU increased by 2.2% and 9.3%, respectively. In the recognition of forest elements, the Precision index increased by 6.8% and 14.5%, Recall increased by 6.8% and 13.8%, and IoU increased by 6.7% and 14.0%, respectively. In the identification of lake elements, the Precision index increased by 4.5% and 6.0%, Recall increased by 4.6% and 5.8%, and IoU increased by 4.3% and 5.7%, respectively. The improvement of forest identification accuracy was particularly obvious.

**Figure 14:**
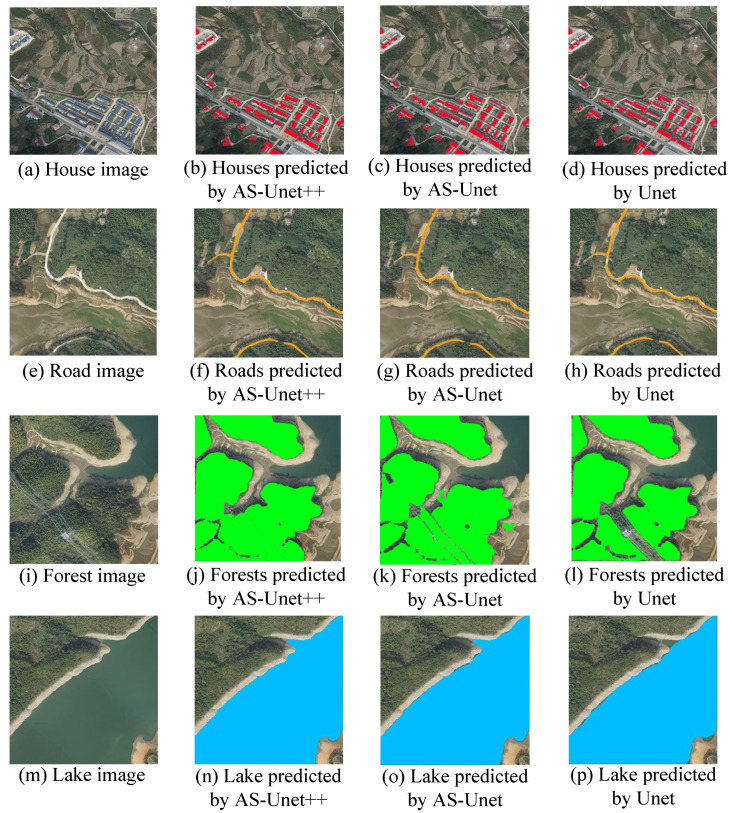
Segmented images predicted by AS-Unet++, AS-Unet, and Unet.

Figure 14 shows a comparison of the predicted segmentation images of houses, roads, forests, and lakes achieved by the three networks.

As can be seen from Figure 14, although Unet is able to recognize the corresponding elements, there is still some misrecognition and omission. In the recognition of houses, a small number of roof pixels are incompletely recognized due to the difference in light received by different surfaces of the roof. In the recognition of roads, there are omissions in the recognition of roads with small widths. In the recognition of forests, the segmentation interference of the power lines at the lower right side leads to the leakage of recognition of the surrounding pixels. There is leakage recognition in the curved part of the lake edge.

Compared with Unet, AS-Unet significantly improved the recognition and segmentation of various elements. In the recognition of houses, the missing recognition phenomenon of Unet has been improved, but there are still a small number of pixels missing recognition in places with large differences in house lighting, which leads to incomplete recognition of all house pixels. In the identification of roads, the phenomenon of misidentification of banded wasteland similar to roads has been significantly improved, but the problem of the missing identification of roads with small widths still exists. In the recognition of forest, the missing recognition is obviously improved, but there is also a phenomenon of misidentifying grassland as forest. In lake recognition, the edge with a complex shape can be segmented correctly, and the performance is obviously improved.

The segmentation effect of AS-Unet++ is improved compared with both Unet and AS-Unet. In the recognition of houses, AS-Unet++ can identify the houses in the figure more accurately. Moreover, there is no missing recognition phenomenon like Unet caused by differences in lighting for a single house. In the road identification, the problem of road leakage identification with small widths can be solved and the banded wasteland similar to the road is not misidentified. In the forest identification of AS-Unet++, the missing identification phenomenon caused by power lines in the lower right is solved, so that the identification area is larger. In the recognition of lakes, the edges with complex shapes can also be correctly segmented.

The Precision, Recall, and IoU of the test sets of each network for road elements, forest elements, and lake elements are shown in Table 6.

The MIoUs of AS-Unet++, Unet, and AS-Unet in the test set were 90.2%, 80.5% and 85.5%, respectively.

It can be seen from the above data that AS-Unet++ is superior to Unet and AS-Unet in each index of the test sets. Compared with AS-Unet and Unet, the MIoU of AS-Unet++ increases by 4.7% and 9.7%, respectively. In the identification of housing elements, the Precision index increased by 3.3% and 7.0%, the Recall index increased by 3.5% and 7.9%, and the IoU index increased by 3.4% and 7.5%, respectively. In the recognition of road elements, Precision index increased by 2.0% and 9.0%, Recall index increased by 2.6% and 9.5%, and IoU index increased by 2.6% and 9.8%, respectively. In the recognition of forest elements, the Precision index increased by 7.7% and 14.9%, Recall increased by 7.5% and 14.5%, and IoU increased by 7.4% and 14.9%, respectively. In the recognition of lake elements, the Precision index increased by 5.1% and 6.6%, Recall increased by 5.4% and 6.5%, and IoU increased by 5.3% and 6.6%, respectively.

(3) **Comparison of AS-Unet++, CE Loss, and Conventional Data Enhancement (CDE)**

AS-Unet++, CE Loss, and CDE are compared in the training sets and test sets to evaluate the method of this paper by further comparative experiments.

**Figure 15:**
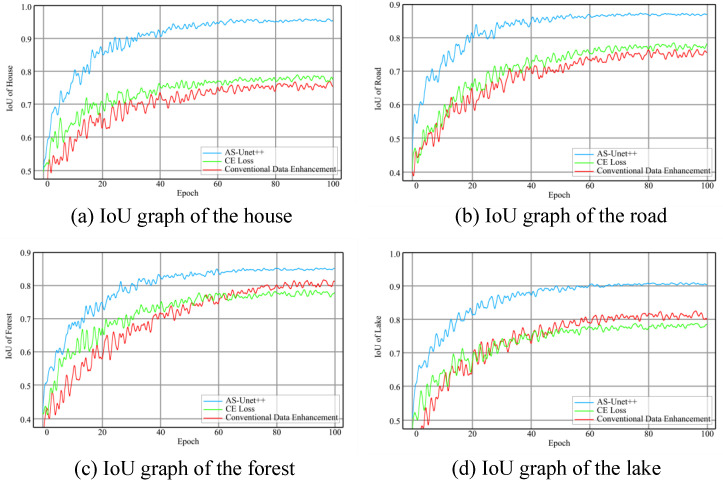
IoU graphs during training of AS-Unet++, CE Loss, and CDE.

The graphs of IoU in the three kinds of networks during the training of houses, roads, forests, and lakes are shown in the Figure 15.

It can be seen that after training, the MIoU of AS-Unet++, CE Loss, and CDE on verification sets is 88.9%, 78.4%, and 78.7%, respectively.

The Precision, Recall, and IoU of the verification set of each network for road elements, forest elements, and lake elements are shown in Table 7.

From the above data, it can be seen that AS-Unet outperforms the other two networks in all accuracy metrics. Compared with CE Loss and CDE, the MIoU of AS-Unet++ is improved by 10.5% and 10.2%, respectively. In Figure 15, AS-Unet++ has the fastest convergence speed and is much faster than the other two networks in the training of roads, forests, and lakes. In the training process, CE Loss and CDE have larger oscillations, especially in the second half of the iteration of the training process, which is still obvious, compared with which the oscillations of AS-Unet++ are smaller and the performance is better.

In the identification of house elements, the Precision index increased by 18.5% and 20.2%, the Recall index increased by 18.7% and 19.6%, and the IoU index increased by 18.7% and 20.1%, respectively. In the recognition of road elements, the Precision index increased by 10.3% and 11.2%, the Recall index increased by 9.7% and 9.6%, and the IoU index increased by 10.0% and 10.3%, respectively. In the recognition of forest elements, the Precision index increased by 6.1% and 5.9%, the Recall index increased by 5.2% and 4.2%, and the IoU index increased by 5.8% and 4.8%, respectively. In the identification of lake elements, the Precision index increased by 12.1% and 10.6%, the Recall index increased by 10.5% and 8.8%, and the IoU index increased by 11.3% and 9.8%. The improvement of house, road, and lake identification accuracy is particularly obvious.

**Figure 16:**
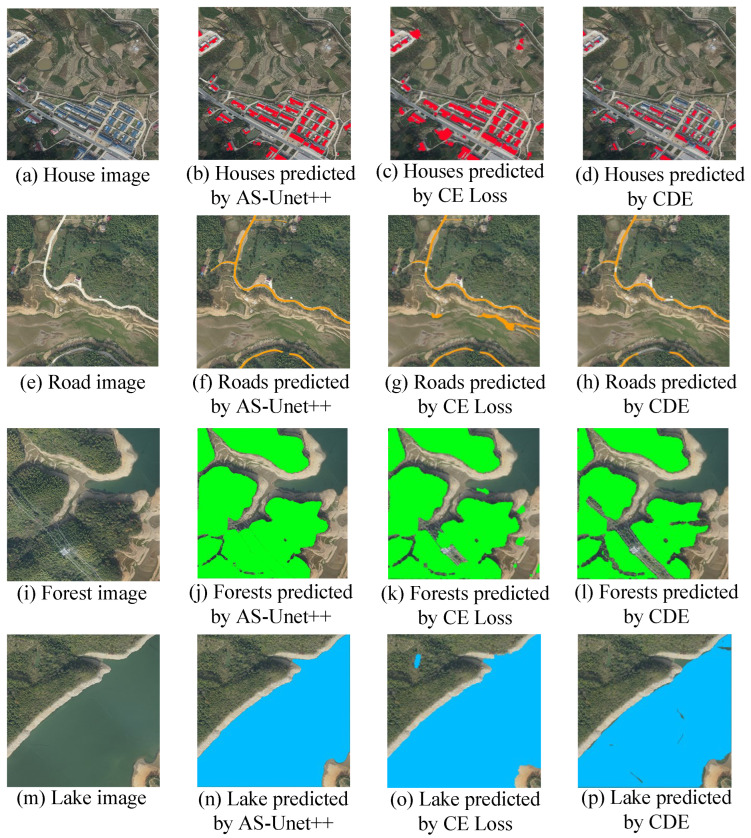
Segmented images predicted by AS-Unet++, CE Loss, and CDE.

As can be seen in Figure 16, all of these networks can essentially recognize the corresponding elements, but there are more significant differences in performance.

Figure 16 shows a comparison of the predicted segmentation images of houses, roads, forests, and lakes achieved by the three networks.

Although CE loss can basically recognize the corresponding elements, there are serious misrecognitions in other non-element parts. In the case of houses, since they are generally rectangular in the image, the network recognizes vehicles and other rectangular elements as houses and also recognizes non-house pixels around the houses as houses. In the recognition of road elements, the roads are distributed in bands, but the network recognizes other non-road elements of the barren land distributed in bands in the image as roads. In the recognition of forest elements, it will recognize some pixels of grass that have a similar color to forest as forest. Lake element identification also suffers from misidentifying a large number of non-lake elements.

In the recognition based on CDE, there are cases where pixels that should have belonged to the corresponding element are not recognized. For example, some houses are not recognized in the house element because the color of different houses varies greatly. In the road element, a road with a small width is not recognized. In the forest element, most of the elements around the lower right side are not recognized because of the interference of power lines. The lake element is not identified because of the color difference of some waters.

AS-Unet++ overcomes the phenomenon that CE Loss misidentifies other similar elements, such as other non-house elements that also present rectangles in the identification of house elements, other elements that also present banded distributions in the identification of road elements, other grasslands similar to forests in the identification of forest elements, and other similar non-lake elements in the identification of lake elements. AS-Unet++ also overcomes the phenomenon of missing recognition in CDE, such as houses with large color differences in house feature recognition, roads with smaller widths in road feature recognition, and forests in forest feature recognition. The interference of the lower right wire is overcome to identify the pixels belonging to the forest, and there is no omission of different colored waters in the lake feature. Compared with the other two networks, AS-Unet++ has better performance.

The Precision, Recall, and IoU of the test sets of each network for road elements, forest elements, and lake elements are shown in Table 8.

The MIoUs of AS-Unet++, CE Loss, and CDE on the test set are 90.2%, 77.9%, and 78.1%, respectively.

It can be seen from the above data that AS-Unet++ is superior to CE Loss and CDE in each index of the test sets. Compared with CE Loss and CDE, the MIoU of AS-Unet++ increases by 12.3% and 12.1%, respectively. In the identification of house elements, the Precision index increased by 20.1% and 21.4%, the Recall index increased by 19.2% and 20.5%, and the IoU index increased by 19.7% and 21.3%, respectively. In the recognition of road elements, the Precision index increased by 10.1% and 8.8%, the Recall index increased by 10.1% and 9.4%, and the IoU index increased by 10.3% and 10.5%, respectively. In the recognition of forest elements, the Precision index increased by 7.8% and 7.0%, the Recall index increased by 5.5% and 4.8%, and the IoU index increased by 6.6% and 5.8%, respectively. In the recognition of lake elements, the Precision index increased by 14.0% and 11.4%, the Recall index increased by 11.9% and 10.0%, and the IoU index increased by 12.9% and 10.8%, respectively.

## 4. Conclusions

In order to achieve the automatic planning of power transmission lines based on remote sensing images, this paper proposes a semantic segmentation method and designs a new AS-Unet++. Compared with the traditional Unet, the ASPP, and the SE Model are added in AS-Unet++, which enhances the neural network’s ability to extract important feature information and capture multi-scale context information. The AS-Unet feature extraction parts of each layer are stacked together, which reduces the amount of training for multiple AS-Unets. AS-Unet++ reduces the number of training parameters compared with the Unet.

Experimental results have shown that the overall recognition accuracies of AS-Unet++ are significantly improved compared to Unet. In the prediction segmentation image, the addition of ASPP improves the edge segmentation, and the addition of the SE model makes the network perform better for the segmentation of houses and roads, which are small elements in the image. In addition, AS-Unet++ can effectively reduce the occurrence of misidentification and missed identification.

Although the method in this paper improves the segmentation accuracy to some extent, the generalization condition is still a great challenge when facing complex and variable remote sensing images, such as elements under different illumination conditions or complex shapes. Future work should be focused on improving the model generalization ability as well as improving the segmentation accuracy even further.

## Figures and Tables

**Table 1:** Environment configuration.

Name	Version
GDAL	3.3.3
segmentation-models-pytorch	0.3.2
torch	1.13.1
pytorch-toolbelt	0.4.1
CUDA	11.1.0

**Table 2:** Parameter setting.

Parameter	Value
Batch size	16
Initial learning rate	0.0001
Learning Momentum	0.9
Weight decay rate	0.001
Total number of iterations	100

**Table 3:** Confusion matrix for classification results.

Reality	Predicted Results	
	**Positive**	**Negative**
Positive	TP	FN
Negative	FN	TP

**Table 4:** Comparison results of different networks for the ablation experiment.

Elements	Valuation Indexs	AS-Unet++	A-Unet++	S-Unet++	Unet++
House	Precision	0.961	0.912	0.932	0.903
	Recall	0.978	0.934	0.955	0.914
	IoU	0.971	0.926	0.943	0.908
Road	Precision	0.862	0.829	0.838	0.798
	Recall	0.877	0.856	0.864	0.807
	IoU	0.872	0.843	0.852	0.803
Forest	Precision	0.850	0.796	0.774	0.753
	Recall	0.859	0.813	0.802	0.766
	IoU	0.854	0.802	0.787	0.759
Lake	Precision	0.907	0.882	0.857	0.852
	Recall	0.917	0.905	0.878	0.863
	IoU	0.912	0.894	0.869	0.858

**Table 5:** Comparison results of different networks on different verification sets.

Elements	Valuation Indexs	AS-Unet++	AS-Unet	Unet
House	Precision	0.953	0.926	0.889
	Recall	0.972	0.940	0.898
	IoU	0.966	0.935	0.893
Road	Precision	0.870	0.845	0.777
	Recall	0.879	0.858	0.783
	IoU	0.874	0.852	0.781
Forest	Precision	0.847	0.779	0.702
	Recall	0.856	0.788	0.718
	IoU	0.851	0.784	0.711
Lake	Precision	0.902	0.857	0.842
	Recall	0.911	0.865	0.853
	IoU	0.905	0.862	0.848

**Table 6:** Comparison results of different networks on different test sets.

Elements	Valuation Indexs	AS-Unet++	AS-Unet	Unet
House	Precision	0.961	0.928	0.891
	Recall	0.978	0.943	0.899
	IoU	0.971	0.937	0.896
Road	Precision	0.862	0.842	0.772
	Recall	0.877	0.851	0.782
	IoU	0.872	0.846	0.774
Forest	Precision	0.850	0.773	0.701
	Recall	0.859	0.784	0.714
	IoU	0.854	0.780	0.705
Lake	Precision	0.907	0.856	0.841
	Recall	0.917	0.863	0.852
	IoU	0.912	0.859	0.846

**Table 7:** Comparison results of AS-Unet++, CE Loss, and CD on different verification sets.

Elements	Valuation Indexs	AS-Unet++	CE Loss	CDE
House	Precision	0.953	0.768	0.751
	Recall	0.972	0.785	0.776
	IoU	0.966	0.779	0.765
Road	Precision	0.870	0.767	0.758
	Recall	0.879	0.782	0.783
	IoU	0.874	0.774	0.771
Forest	Precision	0.847	0.786	0.788
	Recall	0.856	0.804	0.814
	IoU	0.851	0.793	0.803
Lake	Precision	0.902	0.781	0.796
	Recall	0.911	0.806	0.823
	IoU	0.905	0.792	0.807

**Table 8:** Comparison results of AS-Unet++, CE Loss, and CD on different test sets.

Elements	Valuation Indexs	AS-Unet++	CE Loss	CDE
House	Precision	0.961	0.760	0.747
	Recall	0.978	0.786	0.773
	IoU	0.971	0.774	0.758
Road	Precision	0.862	0.761	0.774
	Recall	0.877	0.776	0.783
	IoU	0.872	0.769	0.767
Forest	Precision	0.850	0.772	0.780
	Recall	0.859	0.804	0.811
	IoU	0.854	0.788	0.796
Lake	Precision	0.907	0.767	0.793
	Recall	0.917	0.798	0.817
	IoU	0.912	0.783	0.804

## Data Availability

The data used to support the findings of this study are available from the corresponding author upon request.
